# Resilience: A Critical Appraisal of the State of Research for Business and Society

**DOI:** 10.1007/s41471-022-00151-x

**Published:** 2022-12-19

**Authors:** Tine Buyl, Thomas Gehrig, Jonas Schreyögg, Andreas Wieland

**Affiliations:** 1 Tilburg U., Tilburg, The Netherlands; 2 U. Wien, Wien, Austria; 3 U. Hamburg, Hamburg, Germany; 4 Copenhagen Business School, Copenhagen, Denmark

## Introduction

Establishing resilience seems to have become the great challenge of the new millennium. The concept of resilience has been repeatedly brought to prominence by the fundamental crises of the early 21st century. The Great Financial Crisis (GFC) in 2007/8 brought to the fore the resilience of an interconnected global financial system. The ongoing COVID-19 pandemic that started in 2019 was the motivating event for initiating this Special Issue Resilience. This pandemic caused major multi-layered challenges that span across national health care systems, global supply chains, and beyond. Another event that underlines how resilience spans the boundaries of different systems is Russia’s war against Ukraine, which started in February 2022. This war has substantial economic consequences with fossil energy becoming a key weapon against countries supporting the victim of the Russian aggression, thus dramatically increasing the focus on the lack of resilience of European countries. Almost unnoticed because of these developments closer to home, the resilience of the Earth’s climate system is increasingly spiraling out of control.

In all these events resilience, or the lack of it, has become a key issue of economic, ecological, societal, and political debates. How can society cope with slow but fundamental changes, as well as sudden shocks, and how can or should it deal with them? How effectively are society and its constituent members prepared for these events, some of them formerly being almost unimaginable? Are we prepared for future major surprises or shocks—or, how can we prepare for such events?

The term resilience is on everyone’s lips these days, but it is—perhaps as a result of its widespread use—ambiguous, which is why we consider it necessary to clarify our understanding of the term from the very beginning. We define resilience as “the capacity [of a system] to persist, adapt, or transform in the face of change” (Wieland and Durach 2021, p. 316; see also Duchek 2020). This definition applies to individual firms, but also to institutions, and even to society at large. It implies certain degrees of preparedness, risk avoidance and flexible response capabilities and, notably, goes beyond “bouncing back”.

This definition already contains a long-term perspective. Individuals, firms or institutions exiting markets, supply chains, or societies during an exogenous shock are constantly facing change. The *persist* element of our resilience definition assumes that systems (firms, for example) should avoid failure and, thus, strive for stability and control ensuring operational continuity at any time. However, the *adapt* element of the definition highlights that systems generally need to respond to systemic environment changes by changing themselves. Finally, the *transform* element of the definition points to the fact that sometimes fundamental changes of the narratives and identities of systems are needed to be resilient in the long run. This element of the definition also implies that to be resilient means to take a holistic view that simultaneously acknowledges changes across the multiple levels of our world (planetary, societal, economic, individual levels etc.).

On a societal level, resilience is a public good and, therefore, is most efficiently supplied by public firms or the government (Bös 1981, 1989; Hellwig 2022). This applies specifically to critical infrastructures, health care, transportation, security and supply of critical materials. Infrastructures including appropriate technical and staff equipment require long-term investments. As such these key elements of resilience are in constant danger of being undersupplied in liberal societies with limited tenure of decision makers, because regular contests for office effectively shorten planning horizons. At the same time, and for similar reasons, business relations with uncontested monopolists or autocrats pose specific long-term challenges for resilience.

## Specific Challenges

While resilience is a universal concept, it can be illustrated most easily in specific contexts. A good start is the financial sector, where specific regulation to render the global financial system more stable has failed in making it more resilient. After explaining the main issues of resilience in the financial system, we will continue to discuss more general challenges as the evolved subsequently over time, first in the health sector, then in supply chains, and ultimately for organizations in general.

- **Resilience of the Financial System**

In 1988 the so-called Basel process of capital regulation was triggered in order to render the global financial system safe and sound while maintaining a level-playing field. In the run-up to the financial crisis of 2007/8, however, measures of systemic risk exposure (SRISK) have been increasing for the largest European banks (Gehrig and Iannino 2021) suggesting a steady decline in resilience of the largest and systemically most risky institutions. The crisis itself shifted the regulatory focus from micro-prudential regulation to more system-oriented macro-prudential regulation (Freixas et al. 2015), specifically from the safety of individual institutions towards the safety and soundness of the overall financial system. But still, until the outbreak of the pandemic in 2020 systemic risk exposures of the systemically most relevant European banks did not decline to levels prior to the 2007/8 GFC. Apparently, the political processes did not restore pre-crisis resilience for the most relevant banks that have continued gambling with expected bail-out support in case of insolvency. Interestingly, Scholtens et al. (2019) and Gehrig et al. (2022) document a lower level of systemic risk exposures, and hence a higher level of capitalization and resilience, for banks that reflect a longer planning horizon based on their investments in socially responsibility measures. These findings suggest that bank planning horizon positively contributes to bank capitalization and, ultimately, bank resiliency.

b. **Public Sector and Health**

The concept of resilience has a long tradition in disaster reduction planning which has important overlaps with health care, for example in health emergency planning. However, its application to health care systems and health care organizations is rather new and mainly emerged during the COVID-19 pandemic. During this crisis a fundamental awareness was raised that institutions and health actors should prepare for, recover from and absorb shocks while at the same time maintaining core functions and serving the acute care needs of the population. COVID-19 has overwhelmed many health care systems, each of them in different ways. Some of them were lacking hospital bed capacities, others were lacking staff for beds and again others were not able to distribute vaccines timely and effectively. This highlighted the need to better understand the elements of effective organizational and institutional responses to exogenous shocks (Haldane et al. 2021).

Resilient health care systems are typically developed around the following elements: governance, financing (incentives for providers and individuals, for example), health service delivery (hospitals, for example), public health functions (monitoring of diseases and isolating individuals, for example), health information systems, health workforce, medicines and supplies. For each of those elements scientific literature emerged since the beginning of the pandemic and sometimes before. One stream of literature developed models for forecasting demand for hospitals and public health institutions in crisis and chaotic environments (Morton et al. 2021). Another stream dealt with decisions such as bed allocation in health care organizations in situations with scarce resources emerging in times of crisis (Melman et al. 2021). While many papers were published on resilient communication of government and organizations (Bui et al. 2019) and citizens responses (Neumann-Böhme et al., 2020), rather few papers dealt with the increasing resilience of supply chains, for example for vaccines, or medical supplies necessary to produce test kits or pharmaceuticals (Kazancoglu et al., 2022). Moreover, increasingly a stream on resilient health care workforce planning is expanding (Kuhlmann et al. 2021).

Many health care organizations are public sector organizations, but there is also a research stream on resilience of public sector organizations beyond health care. This stream on resilience in public organizations, with a frequent focus on municipalities, also received a boost by the pandemic (Clement et al. 2022). One topic among others is how municipalities can develop strategies to continue to provide services, for example, issuing ID-cards. In this context, strategies towards digitization of public services is of key interest to increase resilience (Shen et al. 2022).

c. **Supply Chain Resilience**

The discussion about resilience in the literature on supply chain management goes back a long way. Interestingly, it emerged at a time when discussion of a related topic—supply chain risk management (SCRM)—was reaching its limits. The idea of SCRM was to transfer the processes and techniques of corporate risk management from the company system to the supply chain system. This included the idea of simply adding supply chain risks (specifically, supply-side and customer-side risks) to risk categories that previously contained only firm-internal risks. However, it soon became evident that the supply chain as a system is inherently far more complex (see Azadegan and Dooley 2021; Ateş et al. 2022) than the company with its more or less closed system properties. In particular, it became clear that high-impact/low-probability risks were often overlooked already during the risk identification phase of SCRM (Pettit et al. 2010). Here resilience appeared as a complementary approach.

Early contributions to the supply chain resilience literature implicitly took the *persist *perspective, proposing that a supply chain is resilient if it is able to bounce back to its original state. Sheffi and Rice (2005), for example, provided an important impetus to this debate, who used the resilience concept of engineers’ material and transferred it from materials to supply chains. Simchi-Levi et al. (2018) developed the notion that both a time-to-survive and a time-to-recovery can be calculated for the various nodes of a supply chain (for example, ports, warehouses, factories) to determine bottlenecks and thus increase resilience. Hereby it is assumed that a decision maker can take total control of the entire supply chain like an engineer can take total control of the system they are designing or optimizing (for example, a subway system). To say this differently, these contributions implicitly assume that a supply chain is an engineerable system that can and should be controlled and that should be kept in a stable state.

In a recent contribution Gehrig and Stenbacka (2022) argue that strategic investments in multi-sourcing maybe a forward looking strategy designed to avoid bottleneck related hold-ups or even wider systemic amplifications as recently witnessed in the European gas markets. In this specific context they argue that investments in LNG terminals can be seen as a protection against strategic risk in bilateral economic relations, even when those terminals are not actively used.

Recently, the engineering view has been increasingly supplemented by contributions from ecological and social-ecological literature (Wieland 2021). A supply chain can certainly be interpreted as an engineerable system, but this overlooks the fact that a supply chain is primarily a socially constructed system for which social science assumptions appear much better suited in many situations. It contains meanings, identities, and truths that, under changed circumstances, soon become meaningless, outdated, and untrue. Sticking to the status quo or trying to bounce back to the original situation can, therefore, often appear to be the opposite of resilient. It is thus logical that the supply chain resilience literature is increasingly focusing on adaptability and transformability and that the persistence of the supply chain only appears as a goal that makes sense under short-term conditions.

d. **Organizational Resilience**

In the current highly uncertain and volatile times, resilience at the organizational level—defined as “the ability of organizations to anticipate, avoid, and adjust to shocks in their environment” (Ortiz-de-Mandojana and Bansal 2016, p. 1615)—has become all the more relevant. Resilient organizations anticipate and prepare for unexpected disruptions in their environment, respond to such disruptions when they occur, and adapt their processes and structures in order to survive and prosper (Sutcliffe and Vogus 2003). Academic interest in organizational resilience is relatively new, but has grown steadily in the last years, and especially after the occurrence of the 2007/8 GFC. Early work on resilience in an organizational context generally focused on resilience at the individual employee level, building on research in (positive) psychology (DesJardine et al. 2019). However, lately a smaller but growing body of work has emerged identifying antecedents and outcomes of resilience at the organizational level (Duchek 2020). This recent literature stream is still rather fragmented (Linnenluecke 2017), building on insights from a variety of different research areas, such as organizational reliability, adaptability of business models, and crisis management, and even related areas such as corporate social responsibility (CSR), risk management, and organizational ambidexterity.

Based on prior organizational resilience research, two main challenges are especially apparent for (future) scholars in this field: (1) how to conceptualize and operationalize the concept of organizational resilience and (2) how to unveil the main organizational capabilities that constitute resilience, as well as conditions for their development. First, organizational resilience is in essence a latent concept, which cannot be observed and measured directly. Hence, prior research has often inferred resilience indirectly and retrospectively, by studying the changes invoked in other organizational outcomes after an exogenous shock, such as the pattern of stock prices or the growth in sales (DesJardine et al. 2019). Such retrospective analysis after a shock is informative, but it makes it hard to fully uncover how organizational resilience may be achieved in practice.

The latter is closely connected to the second challenge: we need more knowledge about and understanding of organizational capabilities for resilience—specifically, how resilience at the organizational level precisely works, how it is achieved, and how it can be developed (Duchek et al. 2020). Investigating such organizational capabilities introduces a process perspective on resilience. We may need new research designs that move away from retrospective analyses, towards a more qualitative and ethnographical research design, to fully capture the very complex, path dependent, and socially embedded nature of organizational resilience capabilities. More generally, in-depth research on antecedents and drivers of organizational resilience is highly needed. Especially the role of the organization’s (strategic) leaders seems to be essential in this respect (Buyl et al. 2019, for example).

## Future Challenges

Although research on resilience has flourished recently motivated by the pandemic and the war crisis in Ukraine, many research questions remain unaddressed or require further attention.

In the field of health care the issue of resilient allocation of scarce human resources, for example, for hospitals, to provide buffers for unforeseen events, increasingly receives attention in the public discussion. However, in the scientific literature this research question is still not sufficiently addressed from a planning perspective as well as from an organizational perspective. In particular, empirical quantitative or qualitative research on resilient workforce planning is lacking. As already mentioned, there is a need for papers on increasing resilience of supply chains, for example, for vaccines or medical supplies necessary to produce test kits or pharmaceuticals. One key question is here how supply chains can be planned considering resource constraints but also uncertainties, for example geo-political developments.

For public organizations further research is needed on organizational strategies to continue to provide services being resilient to exogenous shocks. Similar to health care resilient work force planning in the face of demographic changes require further attention. Resilient processes through automatization and digitization in all kinds of public sector organizations are further topics providing potential for research papers.

Supply chain resilience faces numerous challenges in business reality. Due to the complexity of global supply chains, there is a high degree of dependency between all the actors involved. Failure to deliver a single part upstream in the supply chain can ultimately result in the inability to build complex end products. However, there is often a lack of transparency in supply chains and companies often do not know who the suppliers’ suppliers actually are. Social and ecological aspects are particular challenging. Companies are often not in a position to know for sure whether questionable practices such as child labor are present in their supply chain, and in order to be able to calculate the emissions of a product, and to be able to calculate the emissions of a product, the end producer would have to know all the emissions along the entire supply chain. In reality, this is difficult to achieve due to the systemic characteristics of a supply chain and its dynamic interactions with the rest of the world.

In the area of organizational resilience, one major challenge for future scholars will be to unravel which factors and characteristics of organizations and their business models can distinguish highly resilient from lowly resilient ones (Chen et al. 2021). An important precursor for this seems to be organizations’ capacity to combine requirements for both stability and flexibility (as both are essential to achieve resilience). This bears close resemblance to the concept of ‘organizational ambidexterity’, which refers to organizations’ ability to simultaneously manage their current business demands and be adaptive to changes in the environment (Gibson and Birkinshaw 2004). In that respect, the role of organizational leaders is not to be underestimated, as they need to be able to cope with the paradoxical demands related to resilience. Scholars have recently found that specific managerial attributes can be linked to organizational resilience. For instance, resilience to systemic shocks seems to be impaired when organizations have a more narcissistic (Buyl et al. 2019) or greedy CEO (Sajko et al. 2021). On the other hand, leaders’ long-term orientation was found to increase organizations’ long-term strategies such as innovation and stakeholder relationships, boosting organizational resilience (Flammer and Bansal 2017). Finally, building organizational resilience also requires awareness of potentially vulnerable critical functions, and the impact of potential shocks on them. Engagement with critical stakeholders—both internal (employees, for example) and external (suppliers and customers, for example)—is essential in this respect (DesJardine et al. 2019).

## Some Preliminary Answers

Several scholars have started to address these current challenges in resilience research. In this SBUR Special Issue, we have included the following contributions that, all in their own way add to our understanding of resilience, its antecedents, and its implications:

For the health care sector, Behrens et al. (2022) develop a concept of resilience particularly suited to health care. Particularly in the health care sector there is a large uncertainty about the definition and function of a resilience concept from an organizational perspective. Thus, the authors synthesize and augment essential resilience concepts from a large body of literature to identify five critical dimensions of resilience in health care. Based on this, they develop an innovative, integrated resilience path concept. These paths are characterized by organizational core capabilities available within a given health care system and evaluated for two distinctively different events—adverse events and planned interventions. The resilience dimensions are mostly illustrated based on examples from the British National Health Service (NHS).

During the pandemic nonprofit organizations (NPOs) have played a major role in many countries in responding to the crisis and in mitigating its effects on populations as well as organizations. NPOs are different from many other organizations since they are strongly mission driven. Stötzer et al. (2022) shed light on how NPOs were coping with disruptive extreme context and which mechanisms they developed in order to maintain service delivery to serve the vital needs of clients. Based on a qualitative content analysis interviewing nonprofit executives, the paper explores the impact of the pandemic on Austrian NPOs active in health and social care in terms of contextual challenges faced. The authors were able to identify important resilience mechanisms (specifically, resilient behavior, resources and capabilities) in NPOs. They differentiate those mechanisms according to different contexts, specifically tasks context, temporal context, physical context and social context.

The pandemic has also shown how relevant it is to think decisions not only from the point of view of one’s own organization, but also from the point of view of the supply chains in which this organization is embedded. This perspective is taken in Trunk and Birkel (2022). The authors focus on the supply chains of small- and medium-sized enterprises, which have often been particularly affected by the pandemic. Based on data from a multiple case study, resilience theory is linked to the relationships between eight such enterprises as well as their suppliers and customers. The authors demonstrate why and how such companies have been able to anticipate and manage the pandemic: “[T]hose companies that made the largest investments in the relational aspects of their partnerships while safeguarding product and financial flows through contracts performed best.” It becomes clear that building relationships matters in order to achieve a high level of supply chain resilience.

Radic and coauthors (2022) focus on resilience at the business model level, indicating the extent to which organizations can maintain and recover their value proposition when faced with an unexpected event. The authors extend the current state-of-the-art by developing a framework for business model resilience, including 11 factors that typify the resilience of an organization’s business model. They also explicitly identify the connections of the framework to indicators of organizational performance, making it a practically relevant tool for managers and decision-makers to assess and improve the resilience of their organizations’ business model.

Möller et al. (2022) connect organizational resilience to the concept of contextual ambidexterity, arguing that successful (resilient) organizations are able to combine the paradoxical activities of managing current business demands as well as adapting to changes in the environment. The authors extend our understanding of contextual ambidexterity and its evolution by examining the impact of control levers (belief and boundary systems) and control context (social and performance management context). They find that a focus on formal boundary systems and an informal social context are positively related to contextual ambidexterity, which is, in turn, positively associated with firm performance.

Förster et al. (2022) also embrace the innate paradoxes in resilience. These authors have a specific focus on the cognitive and behavioral attributes of leaders that can facilitate resilience. They show that when facing a crisis, leaders can apply different paradoxical behaviors to cope in an effective way with the disruption and help their organization in navigating through the event. Using an inductive analysis, the authors identified six pairs of paradoxical behaviors that increase leaders’ ability to effectively deal with the contradictions related to crisis situations, and hence that lead to higher organizational resilience.

Analogously, Weis and Klarner (2022) also zoom in on the role of organizational leaders; more specifically, these authors focus on the role of the organization’s CEO. They provide a fine-grained understanding of organizational resilience by unveiling how CEOs prepare for and adapt to unexpected events on the basis of their ‘future temporal depth’—that is, the temporal distance into the future that the CEO generally takes into consideration when contemplating future events. The authors find that a CEO with a longer future temporal depth is associated with less severe economic losses, but a longer recovery time after the occurrence of a shock. If such a CEO is surrounded by a functionally diverse team, the losses are less severe. In addition, prior experience with organizational crises reduces recovery time.

Preparation for potential disruptive events is also central in Kampmann and Pedell’s (2022) study, which focuses on risk communication as a key factor for developing organizational resilience capabilities, through raising the awareness of vulnerable critical functions and processes and the impact of potential events. The authors examine the influence of the form of risk communication—storytelling versus statistics—on the level of investment in resilience-promoting activities. They found that individuals invest more in a resilience-promoting activity when risk communication is in the form of a story. Furthermore, they explore the effects of a time gap in the risk communication and individuals’ preferences for risk and numbers.

In a similar vein to Trunk and Birkel (2022), Bock et al. (2022) identify the importance of drivers of pro-social behavior fostering the sharing of scarce resources in periods of distress. They document that empathy-elicited altruism may play an even larger role than explicit formal incentives in order to support cooperative resource sharing. Therefore, they advise policy makers to enhance community spirit and social inclusion all the way from the lowest to highest echelons of society.

The Special Issue closes with a commentary largely written from the perspective of German economic policy by Pinkwart et al. (2022). In the concluding section this commentary highlights the urgent need of global coordination in setting and enforcing rules to render societies globally more resilient.
